# Testing Relations of Crystallized and Fluid Intelligence and the Incremental Predictive Validity of Conscientiousness and Its Facets on Career Success in a Small Sample of German and Swiss Workers

**DOI:** 10.3389/fpsyg.2016.00500

**Published:** 2016-04-14

**Authors:** Priska Hagmann-von Arx, Jasmin T. Gygi, Rebekka Weidmann, Alexander Grob

**Affiliations:** Department of Psychology, University of BaselBasel, Switzerland

**Keywords:** intelligence, conscientiousness, career success, occupational skill level, income, job satisfaction

## Abstract

This study examined the relation of fluid and crystallized intelligence with extrinsic (occupational skill level, income) and intrinsic (job satisfaction) career success as well as the incremental predictive validity of conscientiousness and its facets. Participants (*N* = 121) completed the Reynolds Intellectual Assessment Scales (RIAS), the Revised NEO Personality Inventory (NEO-PI-R), and reported their occupational skill level, income, and job satisfaction. Results revealed that crystallized intelligence was positively related to occupational skill level, but not to income. The association of crystallized intelligence and job satisfaction was negative and stronger for the lowest occupational skill level, whereas it was non-significant for higher levels. Fluid intelligence showed no association with career success. Beyond intelligence, conscientiousness and its facet self-discipline were associated with income, whereas conscientiousness and its facets competence and achievement striving were associated with job satisfaction. The results are discussed in terms of their implications for the assessment process as well as for future research to adequately predict career success.

## Introduction

Career success is of importance to individuals as well as to organizations, because it has the capacity to contribute to organizational success (Judge et al., 1999). Career success can be defined as the positive work and psychological outcomes, which have resulted from one's work experiences (Seibert and Kraimer, 2001). It can be structured into extrinsic, objective indicators such as occupational skill level and income as well as intrinsic, subjective indicators such as job satisfaction (Judge et al., 1999; Ng et al., 2005). In addition, the term job performance refers to the performance within one's occupation (Schmidt and Hunter, 2004).

Research in psychology has shown great interest in examining predictors of career success. First, intelligence, the “can do” of a person (Guion and Gottier, 1965, p. 151), has been found to be the strongest predictor of career success (Schmidt and Hunter, 2004). Second, the personality trait conscientiousness, the so-called “will do” (Gottfredson, 2002, p. 37) of an individual, has also been established as an important trait for career success (Hurtz and Donovan, 2000; Sackett and Walmsley, 2014). Both predictors are embedded in a recent integrative theoretical model proposed by Schmidt (2014), suggesting that intelligence, foremost crystallized intelligence (that originally required fluid intelligence for its development), as well as conscientiousness exert direct influence on an adult's occupational achievement. Studies have also shown that conscientiousness demonstrates incremental validity for determining career success and job performance above and beyond intelligence (Schmidt and Hunter, 1998; Judge et al., 1999; Avis et al., 2002). This study extends current research in not only examining the incremental validity of conscientiousness in the prediction of career success beyond fluid and crystallized intelligence but also by analyzing the trait conscientiousness as a whole as well as in its separate narrow facets.

Intelligence can be considered as equivalent to the general factor g, superordinate to all cognitive abilities. Intelligence models (Horn and Cattell, 1966) suggest that g can be divided into two separate general factors, namely fluid g (reasoning and problem solving, independent of acquired knowledge) and crystallized g (accumulated information and verbal skills).

Meta-analytical results show that general intelligence is the best single predictor for extrinsic career success—such as occupational level attained—with moderate correlations around 0.50 (Schmidt and Hunter, 2004). Further, meta-analytic results show smaller associations between general intelligence and income ranging from 0.20 to 0.27 (Ng et al., 2005; Strenze, 2007).

General intelligence can also be linked to intrinsic career success such as job satisfaction, although this association seems to vary depending on job complexity or level of occupation. For instance, empirical results show that general intelligence has a small positive effect on job satisfaction when job complexity is high, whereas it has a small negative effect when job complexity is low (Ganzach and Fried, 2012). Likewise, another study found that the association between intelligence and career satisfaction was significantly negative for lower occupational levels such as hourly employees (*r* = −0.30) but significantly positive for higher occupational levels such as managers (*r* = 0.30; Lounsbury et al., 2004).

Conscientiousness is a dimension of the Big Five model of personality (Costa and McCrae, 1995) and has been shown to be one of the most central personality traits associated with outcomes in the working field (Sackett and Walmsley, 2014). A meta-analytic study reported conscientiousness was linked to work performance across several assessed occupational groups with estimated true correlations above 0.20 (Hurtz and Donovan, 2000). Furthermore, meta-analytic results suggest that conscientiousness is weakly associated with income (*r* = 0.07; Ng et al., 2005) and intrinsic career success such as job or career satisfaction with correlations ranging from 0.14 to 0.26 (Judge et al., 2002; Ng et al., 2005).

As conscientiousness is not a unidimensional construct (Costa and McCrae, 1995), it can be assumed that facets of conscientiousness may each contribute a different amount of variance to career success and job performance. For instance, a meta-analysis (Dudley et al., 2006) showed that the conscientiousness facets achievement, dependability, order, and cautiousness differentially increased the explained variance of overall job performance by 1–24% over and above global conscientiousness. In these analyses, occupational type (sales personnel, customer service representatives, managers, skilled, and semiskilled workers) served as a moderator of the relation between conscientiousness and job performance. For example, in managers there was a negative relation between order (i.e., being well-organized and methodical) and job performance, while in skilled and semiskilled workers the association was positive. Furthermore, a recent meta-analysis (Judge et al., 2013) tested a hierarchical framework in which the trait conscientiousness comprised two lower order traits, industriousness and orderliness, proposed by DeYoung et al. (2007). Both lower order traits encompassed three facets of conscientiousness introduced by Costa and McCrae (1995). Industriousness included the facets achievement striving, competence, and self-discipline, whereas orderliness comprised the facets deliberation, dutifulness, and order. Results showed that the broad trait conscientiousness as well as the two lower order traits industriousness and orderliness were related to job performance with correlations ranging from 0.21 to 0.26. The facets contributed differently to the prediction of job performance with achievement striving, dutifulness, and self-discipline showing the highest associations ranging from 0.19 to 0.24. Although, the effect sizes were modest, the authors concluded that the assessment of lower order traits improved criterion-related validity of job performance over that of the broad trait (Judge et al., 2013).

Research results have revealed that intelligence and conscientiousness independently predict career success. Further, studies have also focused on the question of whether conscientiousness provides incremental validity beyond intelligence in predicting career success and job performance (Schmidt and Hunter, 1998; Judge et al., 1999; Avis et al., 2002). For example, Schmidt and Hunter (1998) reported in a meta-analysis that conscientiousness predicted job performance above and beyond cognitive ability with 10% incremental variance explained. However, we know of no study that has separately considered fluid and crystallized intelligence, as suggested in the theoretical model proposed by Schmidt (2014). Furthermore, we know of no study that has analyzed the incremental validity of facets of conscientiousness on career success beyond fluid and crystallized intelligence.

The current study pursued two objectives. First, we examined the association between intelligence (fluid, crystallized) and extrinsic (occupational skill level, income) and intrinsic (job satisfaction) career success. Based on previous research (e.g., Schmidt, 2014), we expected that intelligence and in particular crystallized intelligence would be a positive predictor of career success. Second, we examined the incremental predictive validity of conscientiousness and its facets in predicting career success. We expected that conscientiousness would be positively related to career success, that conscientiousness would explain incremental predictive variance, and that facets of conscientiousness would contribute differentially to the explanation of career success. Finally, as previous research suggests that the association between intelligence and job satisfaction may vary depending on occupational skill level, we additionally investigated the moderating role of occupational skill level in this association. In low occupational skill levels we expected that intelligence would be negatively related to career satisfaction, whereas in high occupational skill levels we expected that intelligence would be positively related to career satisfaction. Our study extends previous research by investigating both fluid and crystallized intelligence as well as conscientiousness and its facets in order to clarify their simultaneous associations with career success.

## Materials and methods

### Participants and procedure

The sample consisted of 121 adults (48 males, 73 females) with an average age of 48.45 years (*SD* = 12.54 years). The recruitment took place as part of the German standardization and validation of the Reynolds Intellectual Assessment Scales (RIAS; Hagmann-von Arx and Grob, 2014). All participants provided written informed consent to participate. The study and the consent procedure were approved by the Ethics Committee of Basel and the study was performed in accordance with the ethical standards laid down in the Declaration of Helsinki. Participants were from Germany (*n* = 49) and from Switzerland (*n* = 72). At the end of the study, participants received a written report on their test results.

### Measures

Intelligence was assessed using the German version of the RIAS. The RIAS is an individually administered intelligence test for persons between the ages of 3 and 99 years standardized in Germany and Switzerland. It is composed of a two-subtest measure of non-verbal intelligence and a two-subtest measure of verbal intelligence, both of which were developed to closely match the constructs of fluid and crystallized intelligence. An overall intelligence score can be calculated from the sum of the T scores of the four subtests. In the current sample, the internal consistency for the overall intelligence score (α = 0.90) as well as for non-verbal/fluid (α = 0.83) and verbal/crystallized (α = 0.91) intelligence was high.

Conscientiousness and its facets (Competence, Order, Dutifulness, Achievement Striving, Self-Discipline, Deliberation) were assessed with the German version of the NEO Personality Inventory-Revised (NEO-PI-R; Ostendorf and Angleitner, 2004). The NEO-PI-R is a self-report inventory containing 240 items, grouped into 30 facet scales, which are hierarchically organized under the five major dimensions of personality (i.e., Neuroticism, Extraversion, Openness, Agreeableness, Conscientiousness). Each facet contains eight items. Responses are made on a five-point scale (1 = *strongly disagree*; 5 = *strongly agree*). In the current sample, the internal consistency for the composite score of conscientiousness was high (α = 0.82). Facet reliabilities were moderate to high with α = 0.65–0.81.

Job satisfaction was assessed using a short German self-report survey (Neuberger and Allerbeck, 1978) containing eight items, which were rated on a five-point scale (1 = *does not apply at all*; 5 = *applies completely*). Example items are “I really enjoy my work.” and “I am always in a rut with my work; nothing can be done about it” (reverse-scored). In the current sample, a moderate Cronbach's alpha value of 0.76 was recovered.

Personal income was assessed with one item: “How high is your gross income per year?” which was answered by *n* = 76 (63%) subjects. To control for cross-country differences in income, participants' income was divided by their country's most recent purchasing power parity to reflect participants' personal purchasing power within their country. We log-transformed the income according to suggestions by Kahneman and Deaton (2010).

Occupational skill level was calculated on the basis of the participants' profession. This information was encoded according to the four skill levels (1 = *unskilled*; 4 = *highly skilled*) distinguished in the International Standard Classification of Occupations (ISCO-08; International Labour Organization, 2008). Descriptive statistics for demographic variables, intelligence, conscientiousness, and career success including mean, standard deviation, range, skew, and kurtosis are shown in Table 1.

**Table 1:** **Descriptive statistics for demographic variables, intelligence, conscientiousness, and career success**.

**Variable**	***N***	***M* (%)**	***SD***	**Range**	**Skew**	**Kurtosis**
Sex (men)	121	(40)	–	–	–	–
Age (years)	121	48.45	12.54	21.67–77.33	−0.275	−0.356
Fluid intelligence	121	101.80	10.66	61–123	−0.363	0.728
Crystallized intelligence	121	103.26	11.19	66–123	−0.806	0.402
Conscientiousness	121	127.65	18.77	77–184	0.213	0.962
Competence	121	22.43	3.63	11–32	0.045	0.814
Order	121	19.69	4.69	4–30	−0.564	0.827
Dutifulness	121	23.82	3.69	15–31	−0.310	−0.069
Achievement striving	121	19.29	4.26	8–31	−0.073	0.298
Self-discipline	121	19.98	5.23	4–32	−0.436	0.176
Deliberation	121	17.39	4.40	6–28	−0.271	−0.544
Occupational skill level	120	3.20	0.89	1–4	−0.627	−0.885
Logged income	76	4.54	0.28	3.66–5.08	0.632	0.653
Job satisfaction	118	2.70	0.64	1–4	−0.740	0.749

### Statistical procedure

All analyses were carried out using SPSS 22.0. As the sample size was rather small and distributions of some of the variables showed deviations from normality, we used bootstrap procedures (Efron, 1979; Chernick, 2008) for all analyses to construct bias-corrected 95% confidence intervals (BC 95%-CI) based on 5000 random samples. When the confidence interval did not include zero, an effect was considered as significant.

Two sets of simultaneous regression analyses were conducted for each of the career success outcomes. In a first set of analyses, we examined the role of fluid and crystallized intelligence as well as the composite score of conscientiousness in predicting (a) occupational skill level, (b) logged income, and (c) job satisfaction, controlling for age and sex. The second set of analyses included fluid and crystallized intelligence as well as the six facets of conscientiousness predicting (a) occupational skill level, (b) logged income, and (c) job satisfaction, controlling for age and sex.

Regarding the association between intelligence and job satisfaction, two sets of moderated regression analyses were conducted following the procedure proposed by Aiken and West (1991) and Cohen et al. (2013) to investigate whether occupational skill level acts as a moderator of this association. Occupational skill levels 1 and 2 were combined because of a small case number for skill level 1. Thus, occupational skill level was a three-group categorical variable (low, middle, and high occupational skill level) and was dummy coded for inclusion in the regression equations (Tabachnik and Fidell, 2007). In a first set of analyses, control variables, fluid and crystallized intelligence, the composite score of conscientiousness, occupational skill level, and the interaction terms between occupational skill level and intelligence variables were entered into the regression equation predicting job satisfaction. In a second set of analyses, control variables, fluid and crystallized intelligence, the six facets of conscientiousness, occupational skill level, and the interaction terms between occupational skill level and intelligence variables were entered into the regression equation predicting job satisfaction. Variables included in the interaction term were centered. If a significant interaction was identified, indicating a moderation effect, then the interaction was graphed by computing predicted values of job satisfaction separately for each occupational skill level at low (−1 *SD*) and high (+1 *SD*) values of intelligence. Analyses of simple slopes were conducted to evaluate whether the slopes of the independent variables were significantly different from zero in each occupational skill level (Cohen et al., 2013). For all variables, z-standardized scores were used such that the reported unstandardized estimates can be interpreted as standardized regression coefficients.

## Results

As shown in Table 2, in the present sample, sex was correlated to logged income (*r* = −0.281, *p* = 0.014), such that men reported a higher income than women. The composite score of conscientiousness was strongly positively correlated to its six facets (*r* = 0.440–0.771, *p* < 0.001). To avoid problems of multicollinearity, the composite score (model 1) and the six facets of conscientiousness (model 2) were entered in separate regression models.

**Table 2:** **Correlations among all variables**.

**Variable**		***N***	**1**	**2**	**3**	**4**	**5**	**6**	**7**	**8**	**9**	**10**	**11**	**12**	**13**
1	Sex (0 = Men, 1 = Women)	121	1												
2	Age (years)	121	0.106	1											
3	Fluid intelligence	121	−0.188	0.087	1										
4	Crystallized intelligence	121	−0.092	−0.060	0.562^***^	1									
5	Conscientiousness	121	−0.059	0.047	0.075	−0.062	1								
6	Competence	121	0.017	0.147	0.288^*^	0.184	0.641^***^	1							
7	Order	121	−0.190	−0.015	−0.127	−0.270^*^	0.732^***^	0.153	1						
8	Dutifulness	121	0.012	−0.031	−0.163	−0.203^†^	0.658^***^	0.219^†^	0.473^***^	1					
9	Achievement striving	121	−0.055	0.006	0.145	0.076	0.686^***^	0.349^**^	0.360^**^	0.338^**^	1				
10	Self-discipline	121	0.009	−0.002	0.007	−0.088	0.771^***^	0.247^*^	0.633^***^	0.445^***^	0.446^***^	1			
11	Deliberation	121	−0.189	−0.121	−0.054	−0.062	0.440^***^	0.180	0.440^***^	0.446^***^	0.279^*^	0.246^*^	1		
12	Occupational skill level	120	0.152	0.028	0.206^†^	0.307^**^	0.095	0.243^*^	−0.093	−0.101	0.187	0.019	−0.060	1	
13	Logged income	76	−0.281^*^	0.159	0.185	0.124	0.362^**^	0.283^*^	0.260^*^	0.062	0.173	0.425^***^	0.119	0.192^†^	1
14	Job satisfaction	118	0.043	0.108	0.091	0.007	0.397^***^	0.383^**^	0.249^*^	0.098	0.290^*^	0.287^*^	−0.056	0.284^*^	0.086

† *p < 0.10*,

* *p < 0.05*,

** *p < 0.01*,

*** *p < 0.001*.

The results of the simultaneous regression analyses are shown in Table 3. In model 1 (including the composite score of conscientiousness) crystallized intelligence was significantly related to occupational skill level (Estimate = 0.306, *SE* = 0.112, BC 95%-CI = [0.082, 0.514]), whereas fluid intelligence showed no significant association with occupational skill level (Estimate = 0.015, *SE* = 0.097, BC 95%-CI = [−0.170, 0.199]). Neither intelligence factors was related to logged income^1^ (crystallized intelligence: Estimate = 0.110, *SE* = 0.135, BC 95%-CI = [−0.157, 0.361]; fluid intelligence: Estimate = 0.029, *SE* = 0.130, BC 95%-CI = [−0.233, 0.267]), or job satisfaction (crystallized intelligence: Estimate = −0.071, *SE* = 0.099, BC 95%-CI = [−0.259, 0.127]; fluid intelligence: Estimate = 0.059, *SE* = 0.089, BC 95%-CI = [−0.106, 0.244]). In model 2 (including the six facets of conscientiousness), the results regarding crystallized and fluid intelligence predicting career success were comparable to those in model 1.

**Table 3:** **Regression analyses of fluid and crystallized intelligence, conscientiousness (model 1) and its facets (model 2) predicting career success, controlling age and sex**.

**Predictor**	**Occupational skill level (*****n*** = **120)**			**Logged income (*****n*** = **76)**			**Job satisfaction (*****n*** = **118)**		
	**Estimate**	***SE***	**BC 95%-CI**	**Estimate**	***SE***	**BC 95%-CI**	**Estimate**	***SE***	**BC 95%-CI**
**MODEL 1**									
Age	0.074	0.084	[−0.089, 0.228]	0.184	0.117	[−0.042, 0.427]	0.112	0.090	[−0.064, 0.293]
Sex	0.181	0.089	[0.002, 0.361]	−0.253	0.100	[−0.445, −0.061]	0.055	0.091	[−0.137, 0.252]
Fluid intelligence	0.015	0.097	[−0.170, 0.199]	0.029	0.130	[−0.233, 0.267]	0.059	0.089	[−0.106, 0.244]
Crystallized intelligence	0.306	0.112	[0.082, 0.514]	0.110	0.135	[−0.157, 0.361]	−0.071	0.099	[−0.259, 0.127]
Conscientiousness	0.080	0.084	[−0.096, 0.254]	0.388	0.128	[0.128, 0.632]	0.365	0.088	[0.193, 0.549]
F of model 1	3.610^*^			4.566^**^			4.337^**^		
R^2^ of model 1	0.122			0.246			0.163		
**MODEL 2**									
Age	0.037	0.084	[−0.124, 0.189]	0.177	0.127	[−0.069, 0.425]	0.056	0.085	[−0.104, 0.200]
Sex	0.175	0.091	[−0.004, 0.353]	−0.292	0.103	[−0.485, −0.102]	0.027	0.093	[−0.157, 0.226]
Fluid intelligence	−0.047	0.099	[−0.239, 0.136]	−0.007	0.135	[−0.263, 0.249]	−0.010	0.099	[−0.192, 0.195]
Crystallized intelligence	0.263	0.111	[0.029, 0.483]	0.085	0.130	[−0.172, 0.315]	−0.118	0.109	[−0.318, 0.098]
Facets of conscientiousness									
Competence	0.213	0.108	[−0.029, 0.437]	0.211	0.130	[−0.059, 0.472]	0.326	0.119	[0.095, 0.589]
Order	0.028	0.121	[−0.210, 0.269]	−0.027	0.150	[−0.301, 0.255]	0.100	0.121	[−0.130, 0.325]
Dutifulness	−0.159	0.119	[−0.398, 0.084]	−0.155	0.132	[−0.425, 0.100]	−0.123	0.097	[−0.310, 0.055]
Achievement striving	0.190	0.119	[−0.029, 0.427]	−0.090	0.151	[−0.372, 0.204]	0.257	0.108	[0.052, 0.478]
Self-discipline	−0.151	0.122	[−0.390, 0.077]	0.584	0.180	[0.246, 0.907]	0.047	0.122	[−0.205, 0.296]
Deliberation	0.000	0.107	[−0.211, 0.189]	0.031	0.126	[−0.206, 288]	−0.178	0.107	[−0.401, 0.022]
F of model 2	2.623^**^			3.644^**^			3.721^***^		
R^2^ of model 2	0.194			0.359			0.260		

*All variables were z-scored prior to analysis so that all estimates can be interpreted as standardized effects and be directly compared to one another. BC 95%-CI = bias-corrected 95% bootstrap confidence intervals*.

* *p < 0.05*,

** *p < 0.01*,

*** *p < 0.001*.

The composite score of conscientiousness (model 1) did not explain additional variance in occupational skill level (Estimate = 0.080, *SE* = 0.084, BC 95%-CI = [−0.096, 0.254]), although it explained incremental variance in logged income^2^ (Estimate = 0.388, *SE* = 0.128, BC 95%-CI = [0.128, 0.632]), as well as job satisfaction (Estimate = 0.365, *SE* = 0.088, BC 95%-CI = [0.193, 0.549]). In model 2, facets of conscientiousness were not related to occupational skill level. The facet self-discipline (Estimate = 0.584, *SE* = 0.180, BC 95%-CI = [0.246, 0.907]) showed a significant positive association with logged income^3^. The facets competence (Estimate = 0.326, *SE* = 0.119, BC 95%-CI = [0.095, 0.589]) and achievement striving (Estimate = 0.257, *SE* = 0.108, BC 95%-CI = [0.052, 0.478]) were significant positive predictors of job satisfaction (Table 3).

Moderated regression analyses were conducted to examine possible interaction effects of intelligence variables and occupational skill level on job satisfaction. In model 1, these analyses revealed a significant crystallized intelligence × occupational skill level interaction for the dummy variable comparing the low with the middle and high occupational skill level (Estimate = −0.255, *SE* = 0.128, BC 95%-CI = [−0.512, −0.017]), as shown in Table 4. In model 2, the interaction was no longer significant (Estimate = −0.219, *SE* = 0.143, BC 95%-CI = [−0.501, 0.072]). The fluid intelligence × occupational skill level interactions for the dummy variable comparing the low with the middle and high occupational skill level as well as for the dummy variable comparing the high with the low and middle occupational skill level did not reach significance, neither in model 1 (low skill level: Estimate = −0.141, *SE* = 0.017, BC 95%-CI = [−0.369, 0.073]; high skill level: Estimate = −0.056, *SE* = 0.092, BC 95%-CI = [−0.250, 0.110]) nor in model 2 (low skill level: Estimate = −0.151, *SE* = 0.133, BC 95%-CI = [−0.400, 0.098]; high skill level: Estimate = −0.051, *SE* = 0.099, BC 95%-CI = [−0.260, 0.126]).

**Table 4:** **Regression analyses of fluid and crystallized intelligence, conscientiousness (model 1) and its facets (model 2), as well as crystallized intelligence × occupational skill level interactions predicting job satisfaction, controlling age and sex**.

**Predictor**	**Job satisfaction (*****n*** = **118)**		
	**Estimate**	***SE***	**BC 95%-CI**
**MODEL 1**			
Age	0.078	0.092	[−0.105, 0.274]
Sex	0.038	0.094	[−0.146, 0.230]
Fluid intelligence	0.069	0.085	[−0.093, 0.241]
Crystallized intelligence	−0.272	0.110	[−0.475, −0.047]
Conscientiousness	0.317	0.089	[−0.137, 0.495]
Occupational skill level			
Low skill level	−0.130	0.118	[−0.364, 0.110]
High skill level	0.198	0.102	[0.003, 0.399]
Crystallized intelligence × Low skill level	−0.255	0.128	[−0.512, −0.017]
Crystallized intelligence × High skill level	−0.115	0.118	[−0.356, 0.107]
F of model 1	4.062^*^		
R^2^ of model 1	0.255		
**MODEL 2**			
Age	0.035	0.084	[−0.132, 0.191]
Sex	0.025	0.099	[−0.179, 0.220]
Fluid intelligence	0.012	0.095	[−0.161, 0.182]
Crystallized intelligence	−0.262	0.129	[−0.501, −0.004]
Facets of conscientiousness			
Competence	0.269	0.128	[0.012, 0.538]
Order	0.063	0.118	[−0.171, 0.317]
Dutifulness	−0.067	0.104	[−0.280, 0.136]
Achievement striving	0.235	0.124	[−0.006, 0.473]
Self-discipline	0.050	0.131	[−0.216, 0.300]
Deliberation	−0.165	0.099	[−0.354, 0.001]
Occupational skill level			
Low skill level	−0.100	0.123	[−0.331, 0.132]
High skill level	0.149	0.110	[−0.068, 0.367]
Crystallized intelligence × Low skill level	−0.219	0.143	[−0.501, 0.072]
Crystallized intelligence × High skill level	−0.061	0.132	[−0.310, 0.195]
F of model 2	3.397^*^		
R^2^ of model 2	0.318		

*All variables were z-scored prior to analysis so that all estimates can be interpreted as standardized effects and be directly compared to one another. BC 95%-CI = bias-corrected 95% bootstrap confidence intervals*.

* *p < 0.001*.

**Figure 1:**
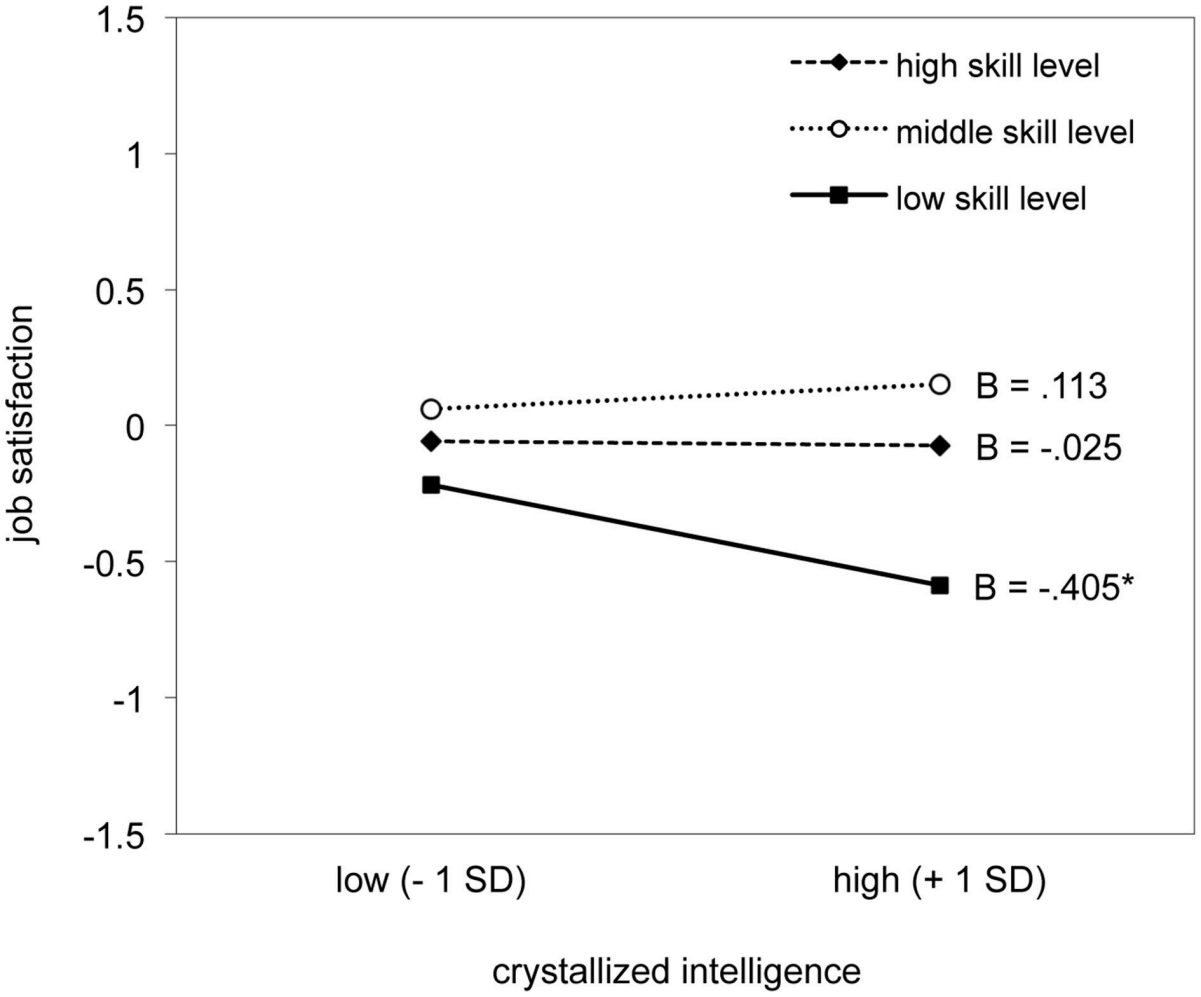
**Crystallized intelligence × occupational skill level interaction in prediction of job satisfaction**. At the lowest skill level, crystallized intelligence was negatively associated with job satisfaction [Estimate = −0.405, *SE* = 0.166, BC 95%-CI = (−0.702, −0.091)], whereas in the middle skill level [Estimate = 0.113, *SE* = 0.229, BC 95%-CI = (−0.287, 0.621)] and high skill level [Estimate = −0.025, *SE* = 0.148, BC 95%-CI = (−0.285, 0.280)] there was no such association. The reported unstandardized estimates can be interpreted as standardized regression coefficients and be directly compared to one another. ^*^p < 0.01.

To further illuminate the significant interaction effect, we conducted single slope analyses. Controlling for age, sex, non-verbal intelligence, and the composite score of conscientiousness, at the lowest skill level, crystallized intelligence was negatively associated with job satisfaction (Estimate = −0.405, *SE* = 0.166, BC 95%-CI = [−0.702, −0.091]) such that lower intelligence was related to higher job satisfaction, whereas in occupational skill level 3 (Estimate = 0.113, *SE* = 0.229, BC 95%-CI = [−0.287, 0.621]) and 4 (Estimate = −0.025, *SE* = 0.148, BC 95%-CI = [−0.285, 0.280]) there were no associations between crystallized intelligence and job satisfaction, as shown in Figure 1.

## Discussion

The current study analyzed the association of intelligence (fluid, crystallized) with extrinsic (occupational skill level, income) and intrinsic (job satisfaction) career success. Furthermore, it examined the incremental predictive validity of conscientiousness and its facets.

Regarding the first hypothesis, whether intelligence is associated with career success, our results revealed that crystallized intelligence is a significant predictor of occupational skill level which is in line with previous studies (Schmidt and Hunter, 2004). Further, this finding corresponds to the integrative theoretical model postulated by Schmidt (2014), which suggests that crystallized intelligence is a major determinant of career success. Crystallized intelligence reflects the knowledge and cognitive skills that a person has acquired through educational and vocational opportunities and thus constitutes expertise. However, the association between crystallized intelligence and occupational skill level was smaller than expected and conflicting with previous research the present study showed no association between fluid intelligence and occupational skill level (Schmidt and Hunter, 2004). Possible explanations may lie in variance restrictions in the present study. The standard deviations for intelligence test scores were lower than in the RIAS standardization sample (*SD* < 15). From previous research it is known that the standard deviation of intelligence test scores increases with decreasing occupational skill level (Schmidt and Hunter, 2004). It may be possible that the small case number of participants with ISCO-08 skill level 1 in the present sample limited the range of intelligence test scores. Thus, restrictions in variance in both intelligence test scores and occupational skill level might have led to attenuated associations between these variables.

Further, intelligence showed no significant relation to income, what is in contrast to previous research (Ng et al., 2005; Strenze, 2007). This result may have emerged because we studied a relatively small sample. Not all participants agreed to answer the open question regarding their income, a recognized problem in social science research (Yan et al., 2010). Based on meta-analytical results (Ng et al., 2005; Strenze, 2007), the expected effects of the relation between intelligence and income were small. For example, according to Strenze (2007) the average association between intelligence and income is 0.20 with a 95% confidence interval of 0.16–0.23. However, estimated “true correlations” calculated in meta-analyses are usually corrected for range restrictions as well as unreliability of the predictor and outcome. Therefore, in a single study, the expected effect may be even smaller. Regarding the current study, *post-hoc* power analysis using G^*^Power (Faul et al., 2007) showed that with a chance above 80% and a 0.05 alpha level we were only able to identify medium-sized effects (Cohen, 1988). Therefore, in the present sample there was not enough statistical power to detect weak associations between intelligence factors and income.

Additionally, we expected that the association between intelligence and job satisfaction varies depending on occupational skill level. This assumption was supported in the present study because results revealed that the association between crystallized intelligence and job satisfaction was moderated by occupational skill level. Crystallized intelligence was a significant negative predictor of job satisfaction in lower occupational skill levels, whereas there was no such association for higher occupational skill levels. The result that in less demanding occupations workers with higher crystallized intelligence report lower satisfaction with their job indicates that workers experiencing cognitive underload are likely to report lower job satisfaction. This is in line with previous research (Lounsbury et al., 2004; Ganzach and Fried, 2012) and might reflect differences in person-work environment fit (Lounsbury et al., 2004). A good person-environment fit can be assumed for workers with high intelligence and high occupational level as they might have the opportunity to develop their potential and therefore be more satisfied with their job. In contrast, a poor person-work environment fit can be assumed for workers with high intelligence and lower occupational level, as they might be restricted in their ability to express their cognitive competence and therefore report a lower job satisfaction. Thus, aligned levels of occupational skill level and intelligence might facilitate job satisfaction.

Regarding the hypothesis, that conscientiousness and its facets explain incremental predictive validity, our results revealed that conscientiousness was significantly positively related to logged income and job satisfaction. These results are in accordance with previous research (Judge et al., 2002; Ng et al., 2005), as well as theoretical assumptions (Schmidt, 2014) and indicate that workers who describe themselves as competent, organized, reliable, self-disciplined, and so on appear to have a higher income and to be more satisfied with their job than workers who describe themselves as being less strong in these characteristics. However, conflicting with previous evidence (Hurtz and Donovan, 2000), in our study conscientiousness was not associated with occupational skill level. From previous research it is known that relations between specific facets of conscientiousness and job performance may differ depending on the investigated profession (Dudley et al., 2006). Our sample included different professions within an occupational skill level such as lawyers, physiotherapists, and university teachers on ISCO-08 skill level 4. Therefore, it may be possible that this heterogeneity masked potential relations within specific professions and led to non-significant associations between facets of conscientiousness and occupational skill level across the whole sample. Furthermore, as the broad trait conscientiousness is a composite score derived from the six facets it is plausible that the relation between conscientiousness and occupational skill level became non-significant when averaging the non-significant effects of the facets (Dudley et al., 2006; Judge et al., 2013).

More detailed analyses regarding the narrow facets of conscientiousness showed that they contribute differentially to income and job satisfaction. In our sample, self-disciplined people had a higher income, whereas people reporting higher competence and achievement striving were more satisfied with their job. These results are in line with previous research showing that facets of conscientiousness differentially increased the explained variance of career success (Dudley et al., 2006; Judge et al., 2013) and thus underline the importance of acquiring a more fine-grained picture of the association between conscientiousness and career success by including facets of conscientiousness.

In our study we measured conscientiousness and its six facets using the NEO-PI-R, which comprises eight items for each facet. Thus, using only particular conscientiousness facets of the NEO-PI-R provides a more parsimonious assessment than the administration of the whole scale, what leads to more efficient testing. However, it remains to be determined by future studies whether questionnaires shorter than the NEO-PI-R such as the short version of the Big Five Inventory (BFI-K; Rammstedt and John, 2005) show comparable associations with career success than the particular conscientiousness facets of the NEO-PI-R.

Future studies might also shed further light on the association between narrow traits and career success. Regarding narrow conscientiousness traits it might be possible that their association with career success varies depending on the different job stages (Woods et al., 2013). For example, Stewart (1999) showed that in newly hired employees for whom demands were novel and possibly not clearly defined, the narrow trait order (e.g., structuring and organizing the work environment, effectively managing time) was more strongly associated with job performance, whereas in senior employees who have mastered the tasks related to their jobs, the narrow trait achievement (e.g., working hard and persistently to achieve goals) was more strongly associated with job performance. However, in a recent study Ganzach and Pazy (2015) showed that temporal changes in career success are mainly driven by intelligence rather than personality traits. Thus, for future studies it would be of interest to further examine predictors of career success at different stages of people's working lives.

Future research might also investigate potential moderators of the relationship between intelligence, conscientiousness, and career success. Regarding the conscientiousness–career success relationship, the meta-analysis conducted by Shaffer and Postlethwaite (2013) revealed a moderating role of job characteristics such that conscientiousness showed a stronger association with job performance in highly routinized jobs whereas this association was weaker in jobs requiring higher levels of cognitive ability. It may be possible that this is due to different ways that conscientiousness is measured: in highly routinized jobs conscientiousness may be rated as arriving at work in a timely manner, whereas in more complex jobs conscientiousness may be related to the skill to manage one's own schedules.

Finally, future research might examine underlying processes potentially affecting the association between intelligence, conscientiousness, and career success to further understand the relation between these variables. For instance, Li et al. (2011) suggest that self-esteem could mediate the relationship between intelligence and career success (i.e., leader role occupancy) because individuals' self-esteem rests on their positive evaluations of their competence.

Limitations of our study include the cross-sectional design, which precludes causal inferences and does not allow testing the direction of the effects between intelligence, conscientiousness and career success. Further, conscientiousness and job satisfaction were both measured using self-reports. In order to reduce common method variance (Podsakoff et al., 2003), which may have had an effect on the relationship between conscientiousness and job satisfaction, future studies might use third-party reports to assess conscientiousness. Also, income was a self-reported variable. Although, this is a common method, the sensitivity of this issue may have led to misreporting (Zinn and Würbach, 2016). Therefore, this variable may not accurately reflect true income and thus may be less valid. In addition, the relatively small sample size regarding income does not allow detecting small effects. Future studies may use closed questions with income brackets to reduce missing data. In addition, our sample consisted of higher-qualified collaborators, thus our results cannot be generalized to unskilled workers. Finally, the study investigated subjects with rather heterogeneous professions. Future studies may shed further light on the association of intelligence, conscientiousness and career success by investigating specific types of professions. However, we consider it a strength of the study that both fluid and crystallized intelligence were assessed using a standardized test and that conscientiousness was analyzed as a broad trait as well as in its separate narrow facets. In addition to a more in-depth look at these predictors, we also examined conceptually distinct aspects of career success with occupational skill level and income representing external aspects and job satisfaction representing an internal aspect of career success. Also, all analyses were controlled for sex, as men reported higher income than women, which is in accordance with the census of Germany (Statistisches Bundesamt, 2014) and Switzerland (Bundesamt für Statistik, 2012).

In conclusion, this study indicates that crystallized intelligence is positively related to occupational skill level and negatively related to job satisfaction in lower occupational skill levels. Hence, examiners may put greater emphasis on crystallized than fluid intelligence when linking intelligence with career success. Beyond intelligence, conscientiousness and its facets—competence, achievement striving, and self-discipline—were differentially associated with income and job satisfaction. To predict career success we therefore propose assessing both intelligence and conscientiousness to combine the “will do” and “can do” aspects of a person.

## Author contributions

PH contributed to the study design, acquisition, analysis, and interpretation of data. Drafted and revised the manuscript, gave final approval, and agrees to be accountable for all aspects of the work in ensuring that questions related to the accuracy or integrity of any part of the work are appropriately investigated and resolved. JG contributed to the acquisition, analysis, and interpretation of data. Drafted and revised the manuscript, gave final approval, and agrees to be accountable for all aspects of the work in ensuring that questions related to the accuracy or integrity of any part of the work are appropriately investigated and resolved. RW contributed to the analysis and interpretation of data. Drafted and revised the manuscript, gave final approval, and agrees to be accountable for all aspects of the work in ensuring that questions related to the accuracy or integrity of any part of the work are appropriately investigated and resolved. AG contributed to the study design and interpretation of data. Revised the manuscript, gave final approval, and agrees to be accountable for all aspects of the work in ensuring that questions related to the accuracy or integrity of any part of the work are appropriately investigated and resolved.

### Conflict of interest statement

The authors declare that the research was conducted in the absence of any commercial or financial relationships that could be construed as a potential conflict of interest.

## Footnotes

^1, 2^The findings did not change substantially when investigating raw income scores.

^3^When investigating raw income scores the facet competence also reached significance (Estimate = 0.258, *SE* = 0.117, BC 95%-CI = [0.017, 0.501]).
